# Internet Behavior Preferences Predict Pathological Internet Use: A Latent Profile Analysis

**DOI:** 10.3389/fpsyg.2021.644623

**Published:** 2021-06-04

**Authors:** Jiangtao Chen, Jinmeng Liu, Gai Zhao, Fanchang Kong

**Affiliations:** ^1^Research Center of Brain and Cognitive Neuroscience, Liaoning Normal University, Dalian, China; ^2^Institute of Developmental Psychology, Faculty of Psychology, Beijing Normal University, Beijing, China; ^3^National Key Laboratory of Cognitive Neuroscience and Learning, Beijing Normal University, Beijing, China; ^4^School of Psychology, Central China Normal University, Wuhan, China

**Keywords:** pathological Internet use, Internet behavior preference, college students, latent profile analysis, person-centered approach

## Abstract

Recent research in the underlying structure of pathological Internet use (PIU) has produced considerable debate among academics, in which a new “person-centered” approach of studying PIU has recently gathered support but produced mixed results. This study used the latent profile analysis (LPA) to estimate the types of PIU in a large sample of college students (*n* = 1,400, aged 17–25 years). Participants provided information on demographics, PIU, and Internet behavior preferences. The adolescent pathological Internet use (APIU), which served as the basis of LPA, was used for searching subgroups that represent participants with PIU. LPA identified the PIU classes, and regressions identified the psychological predictors of class membership. Participants were classified into pathological users, pathological-tendency users, preferential users, and ordinary Internet users. In comparison with pathological Internet users, lower Internet gaming and communication preference were associated with pathological-tendency users, preferential users, and ordinary Internet users. The distinct types of college students belong to each PIU class, suggesting that individual differences may be incorporated into the prevention efforts.

## Introduction

According to the survey report, as of 2019, there were nearly 4.39 billion Internet users worldwide (Kemp, 2019). Moreover, a survey reported that young people were more likely to be online than elders [International Telecommunication Union (ITU), 2018]. In addition, the number of Chinese Internet users had reached 940 million until June 2020, and 18.8% of them were college students [China Internet Network Information Center (CNNIC), 2020]. Earlier studies showed that the prevalence of pathological Internet use (PIU) in China was around 7% (Cheng and Li, 2014), while the prevalence of PIU in Chinese college students was about 11.0% (95% CI: 9.0–13.0%), which is 1.6 times higher than that of the general population. In China, Internet addiction is becoming a significantly growing health problem in college students, which is harmful to their mental health (Shao et al., 2018). Therefore, it is essential to understand what specific patterns of PIU are in college students.

Pathological Internet use, also known as Internet addiction, is a typical excessive Internet use (Caplan, 2002; Meerkerk et al., 2009; Young and Abreu, 2011), including addiction and less severe conditions in which some problems emerge but are still not to the level of full-blown addiction (Lei and Yang, 2007). Currently, there are two main approaches for measuring PIU. One of them is to compel the participant to make a compulsory judgment (i.e., yes or no) on the item, such as pathological use scale (PUS) (Morahan-Martin and Schumacher, 2000). The other one is the Likert scale that allows the participant to make grade judgments, such as the Internet addiction test (IAT) (Young, 1998) and the Chinese Internet addiction scale (CIAS) (Chen et al., 2003). In fact, a compulsive judgment (0–1 score) is more difficult to describe the level of PIU than the Likert scale. IAT is based on the American setting and it focuses on clinical behavior symptoms; however, the degree of fitness of the IAT psychometric properties for the Chinese college students is still unknown. CIAS was designed in the context of Chinese culture. However, it does not include the standards of PIU, such as salience and mood alteration which have attracted more attention in recent studies. Therefore, the content validity of CIAS should be improved. The APIU scale, including cognition and behavior, was developed in Chinese settings (Lei and Yang, 2007) and could measure the level of PIU more comprehensively than CIAS, and it has also been demonstrated to be excellent in validity and reliability for studying the Chinese college students (Li and Zheng, 2010; Sun et al., 2015). Moreover, APIU measured the level of PIU from the perspective of cognition and behavior, which was consistent with the cognitive behavior theory that emphasized the role of cognitions and behavior in PIU of the individual (Davis, 2001).

A large number of previous studies on PIU takes a “variable-centered” approach that focuses on the relationship of PIU with specific outcomes, such as cognitive flexibility, attention set-shifting and bias, impulsivity, and internalizing problems (De Leo and Wulfert, 2013; Forrest et al., 2016; Dieter et al., 2017; Wang et al., 2017). In general, the PIU study about “variable-centered” approach aimed either to describe the characteristics or to determine the impact factors in the entire group. However, few studies focused on evaluating how individuals vary across PIU subgroups, which is important for clinical intervention and individual differences in Internet use. The person-centered approach called latent profile analysis (LPA) for continuous variables instead seeks to identify the homogeneous subtypes of individuals who differ along with their profiles of response on variegated Internet use models (Nylund et al., 2007; Morin et al., 2011).

Numerous recent studies have identified the PIU homogeneous subtypes from “person-centered” perspective. Van Rooij et al. (2011) have promulgated a six-cluster profile model for online gaming addiction and heavy Internet use in a large sample of Dutch school children aged 13–16 years. The findings verified the fitness of the six-class model, in which two of the classes were represented by children who experienced heavy Internet use including excessive online gaming. Similarly, in a study, LPA was applied to explore the subgroups of problematic online social networking use among adolescents and the appropriateness of the three-class model was also demonstrated (Li et al., 2020). However, the above-mentioned studies were restricted to one of the subtypes of PIU so that it was not conducive to our clear understanding. Fortunately, some researchers have paid attention to the class of general Internet use. For example, Mok et al. (2014) applied the latent class analysis (LCA) to classify a total of 463 Korean college students based on the levels of addiction severity and they found that the three-class model was opted to distinguish populations by male or female, which aligned with the findings obtained by the study conducted in 480 Pakistani university students by Hussain et al. (2020). Lee et al. (2018) found that the four-class model was the best fit model among 555 Korean middle-school students. In addition, Rumpf et al. (2014) applied LCA to explore subgroups of Internet-addicted Germans aged 14–64 years, and they found that the six-class model was appropriate. Taken together all the above findings, the classification of PIU was inconsistent in different cultures, in individuals of different ages, or even in small sample sizes.

The antecedents or potential adverse consequences of PIU have been explored from two main perspectives in earlier studies. One of the perspectives is to focus on finding how mental behavior variables play a role in PIU from the perspective of the user, such as self-esteem, personality traits, and loneliness (Tian et al., 2018; Arafa et al., 2019; Koronczai et al., 2019). The other perspective is to explore the causes of PIU from the Internet perspective, such as online activities and Internet anonymity (Leung, 2004; Schehl et al., 2019). However, to our knowledge, few studies examined PIU from the perspective of both the users and the Internet. Therefore, in this study, we applied Internet behavior preference (IBP) to comprehensively investigate PIU from the integrated perspectives. IBP refers to individuals in cyberspace, driven by a certain motivation, who show relatively stable attitudes and behavioral tendencies which reflected the online activities from the perspective of the Internet and personal choice from the perspective of the users (Zhou and Gu, 2008). Many studies indicated that IBPs, such as online gaming (Van Rooij et al., 2010; Rehbein and Mößle, 2013) and social networking preferences (Kuss and Griffiths, 2011), were significantly related to PIU. Some studies showed that PIU could be positively predicted cross-sectionally by gaming preference (Meerkerk et al., 2006; Siomos et al., 2008), directly through Internet social contact preference (Luo et al., 2010), or excessive entertainment and social functions (Li and Chung, 2006). However, earlier studies examined only the relationship between one of the IBPs and PIU, which did not fully reveal the relationship between them. In addition, there is a rapid increasing growth of applications and options for social engagement, and there may exist individual differences among IBP which should be considered in PIU. Therefore, this study hypothesized that various types of IBPs may play a unique role in predicting PIU.

The associations between IBPs and PIU, mainly using variable-centered approaches, described in earlier studies, were quantified by separate models. On the other hand, the person-centered approaches have the advantage of assessing how the PIU behaviors interact with each other and may therefore more comprehensively reflect the complexity of such behavior. On the identification of PIU classes, various individual difference factors can then be used to predict group membership. Understanding how these factors predict classes of PIU may aid in identifying the profiles of PIU of those who are most likely to be engaged and thus provide insight into which factors should be potentially targeted during prevention efforts. Thus, the present data analysis was designed to identify the PIU classes of college students using exploratory LPA and to determine whether gender and IBP characteristics could predict the class membership identified through exploratory LPA.

## Methods

### Participants and Procedure

A total of 1,400 college students (33.71% males, 66.29% females; mean age = 20.24 years, *SD* = 1.52) from different schools across 5 Chinese provinces (i.e., Liaoning, Anhui, Hubei, Henan, and Shandong) participated in this study. The percentage values of freshmen, sophomores, juniors, and seniors were 18.64, 23.43, 24.14, and 33.89%, respectively.

The questionnaires were administrated by distributing the corresponding link to the college students *via* social networking sites (including WeChat and QQ). Informed consent was obtained from all participants. The Institutional Review Boards of relevant universities approved all the study procedures. The participants were informed that the survey was anonymous and voluntary, and the collected data would only be accessible to researchers to maintain confidentiality. The total duration of the online survey was 15 min.

### Measures

#### Adolescent Pathological Internet Use

The APIU, which was developed by Lei and Yang (2007), was used to measure the levels of PIU. APIU comprised 38 items with six dimensions, namely, salience (e.g., “I forget nearly everything else when I am online”), tolerance (e.g., “I find that I increasingly spend time online”), withdrawal symptoms (e.g., “I feel upset when I cannot access the Internet”), mood alteration (e.g., “Going online makes me feel better when I am depressed”), social comfort (e.g., “I feel safer while communicating with others through the Internet”), and negative outcomes (e.g., “I have some difficulty with school performance because I spend too much time on the Internet”). The extent of agreement to each item was evaluated using a 5-point scale (1 = never true to 5 = always true). The Cronbach's alpha coefficients of APIU were reported to be >0.955 among college students in the earlier studies (Li and Zheng, 2010; Sun et al., 2015). In this study, the Cronbach's alpha coefficients were 0.955 for full scale, 0.756 for salience, 0.740 for tolerance, 0.923 for withdrawal symptoms, 0.896 for mood alteration, 0.885 for social comfort, and 0.797 for negative outcomes. In this study, the goodness-of-fit indexes from the confirmatory factor analysis were χ^2^/df = 3,048.686/650, Root-mean-square error of approximation (RMSEA) = 0.051, 90% CI [0.050, 0.053], Comparative fit index (CFI) = 0.900, and Standardized root mean square residual (SRMR) = 0.043. Therefore, the APIU is suitable for college students in this study.

#### Internet Behavior Preference

The IBP, which was developed by Zhou and Gu (2008), was used to measure the levels of the preferences of Internet behavior. The IBP contains 18 items with 3 dimensions, namely, information gaining (e.g., “I spend a large amount of time searching or downloading some materials on learning”), Internet gaming (e.g., “I often play online games; they are thrilling and real-time strategic”), and communication preferences (e.g., “I often use MSN, QQ, WeChat, or other apps to communicate with others”). The respondents were asked to rate whether the content was true for them using a 5-point scale (1 = never true to 5 = always true). The Cronbach's alpha coefficients of IBP were reported to be >0.70 in the earlier study (Zheng and Gu, 2013). In this study, the Cronbach's alpha coefficients were 0.718 for full scale, 0.685 for information gaining, 0.896 for Internet gaming, and 0.610 for communication preferences.

#### Statistical Analysis

The LPA parameters were computed using Mplus 7.1 software (Muthén and Muthén, 1998–2015) and were later used as a base to explain and determine the subgroups that represented APIU. The model that was finally selected by LPA is not only determined by a single indicator, but also based on multiple indicators, such as Akaike information criteria (AIC), Bayesian information criterion (BIC), entropy, Lo–Mendell–Rubin likelihood ratio test (LMRT), bootstrapped likelihood ratio test (BLRT), and the number of parameters (Burnham and Anderson, 2004; Nylund et al., 2007). There are four main criteria to decide on the number of classes. (1) Burnham and Anderson (2004) pointed out that BIC value is better than AIC value. This is a relative measure where a lower BIC value is better. (2) The entropy is the second criterion where high values are preferred (Rumpf et al., 2014; Shin et al., 2018) and the maximum value is 1. This measure is a combination of the posterior probabilities. The posterior probabilities denote how well the respondents are classified into their classes. (3) The LMRT and BLRT are used for determining whether the present solution is significantly better than the previous one, as indicated by *p* < 0.05, e.g., the *k* model is better than the *k*−1 model (Akaike, 1974; Schwarz, 1978; Lo et al., 2001). (4) The simplicity of the model is the final one where the smaller the number of parameters indicated the better the model.

After the identification of the latent classes, the separate multinomial logistic regressions were examined to determine whether IBP predicted the class membership after accounting for demographics. The multinomial logistic regression compares multiple groups through a combination of binary logistic regressions in a model. The data encountered the assumptions for use of multinomial logistic regression, including the use of categorical dependent variables and continuous independent variables, independence of observations, and linear associations among predictors and Internet use variables at the bivariate level (Table 1). The models were built in a step-by-step process. To ensure that the findings from our final model were not due to suppression, we first examined a model (i.e., Model 1) that included only demographics and then examined Model 2 that added each of the IBP dimensions. The predictors of class membership were considered statistically significant when *p* < 0.05.

**Table 1:** Correlation, descriptive statistics, and Variance inflation factor (VIF) for key study variables.

	**1**	**2**	**3**	**4**	**5**	**6**	**7**	**8**	**9**	**10**
1. Gender	–									
2. Information gaining	0.011	–								
3. Internet games	0.012	0.046	–							
4. Communication preferences	0.027	0.162^**^	0.049	–						
5. Salience	0.016	−0.019	0.021	0.023	–					
6. Tolerance	0.048	0.000	0.013	0.032	0.515^**^	–				
7. Withdrawal symptoms	0.142^**^	−0.012	0.045	0.027	0.554^**^	0.728^**^	–			
8. Mood alteration	0.100^**^	0.006	0.060^*^	0.032	0.515^**^	0.528^**^	0.639^**^	–		
9. Social comfort	0.009	0.014	0.047	0.027	0.436^**^	0.565^**^	0.609^**^	0.585^**^	–	
10. Negative outcomes	−0.030	0.032	0.012	0.007	0.545^**^	0.728^**^	0.663^**^	0.493^**^	0.544^**^	–
*M*	–	14.829	15.279	15.772	7.670	11.808	27.696	14.777	14.273	18.468
*SD*	–	3.303	6.033	3.205	2.549	4.063	9.551	4.869	5.258	5.847
VIF	1.059	1.029	1.004	1.029	1.664	2.817	3.013	1.987	1.890	2.516

* *p < 0.05;*

** *p < 0.01*.

## Results

### Latent Profile Analysis of Pathological Internet Use

As shown in Table 2, LPA was identified well with the four-class model of APIU given the significant LMR (*p* < 0.001) and BLR (*p* < 0.001) test results. The reasons for the choice of four-class model were as follows: first, this is a relative measure where a lower BIC value is better. In this study, the lower BIC value of the four-class model suggested that it was appropriate. Second, the entropy of the four-class model was the highest among the models in this study, indicating that the four-class model was the best model. Third, the four-class model was better than the three-class model according to the LMR and BLR. Finally, the fewer parameters of the four-class model denote the simpler structure than that of the five-class and six-class models. For the above-mentioned reasons, the four-class model was finally chosen in this study.

**Table 2:** Model fit information for competing latent class models (*N* = 1,400).

**Number of classes**	**AIC**	**BIC**	**Adjusted BIC**	**Entropy**	**LMRT (*p*)**	**BLRT (*p*)**	**Number of parameters**
1	163939.741	164338.303	164338.303	—	—	—	76
2	150525.128	151128.214	150762.902	0.938	0.000	0.000	115
3	146057.039	146864.650	146375.450	0.942	0.000	0.000	154
4^a^	143909.851	144921.987	144308.898	0.945	0.000	0.000	193
5	143027.012	144243.673	143506.696	0.928	0.029	0.020	232
6	142456.376	143877.562	143016.696	0.920	0.620	0.620	271

*AIC, Akaike information criterion; BIC, Bayesian information criterion; LMRT, Lo–Mendell–Rubin likelihood ratio test; BLRT, bootstrap likelihood ratio test*.

a *Selected as the final model*.

**Figure 1:**
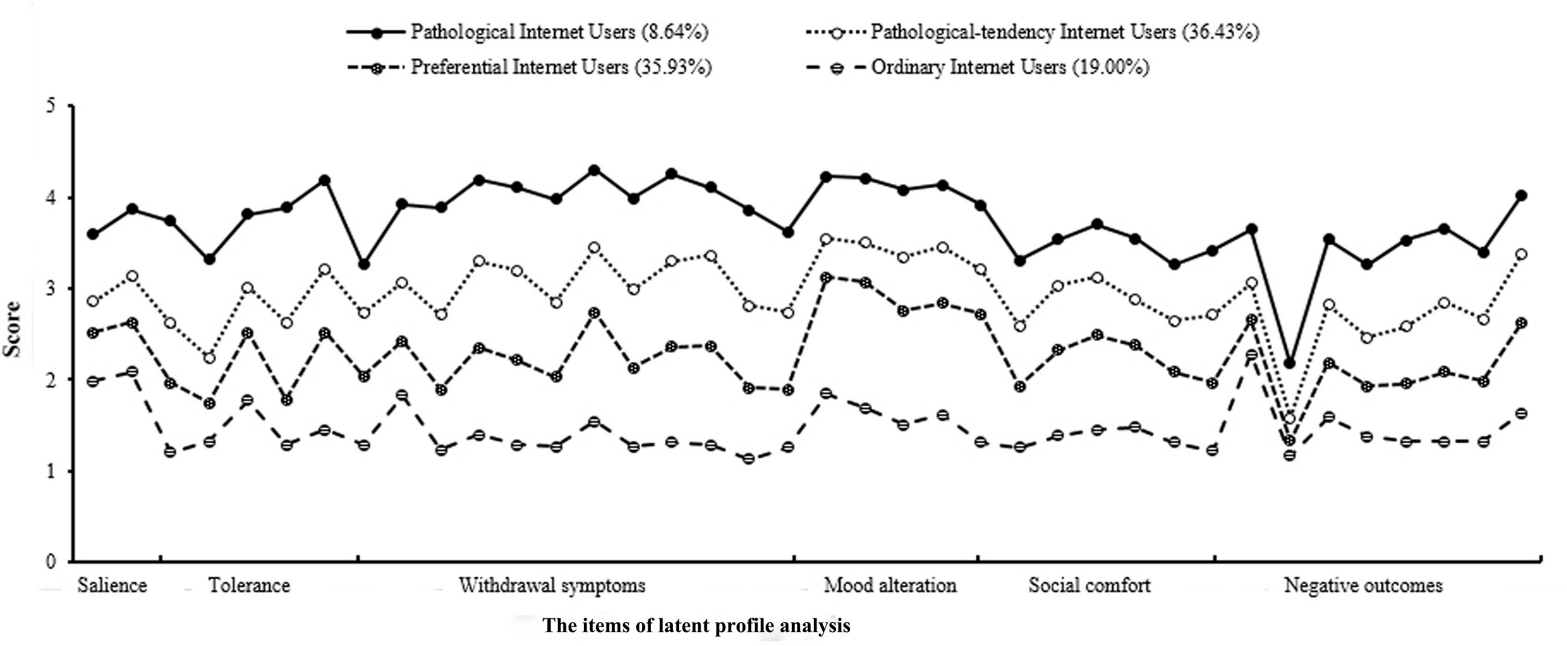
Score plot for the four-class model of college students.

The four classes were represented by the following subgroups: pathological Internet users (class 1: 8.64%, *n* = 121, APIU: *M* = 144.05, *SD* = 10.95), pathological-tendency Internet users (class 2: 36.43%, *n* = 510, *M* = 114.29, *SD* = 7.33), preferential Internet users (class 3: 35.93%, *n* = 503, *M* = 92.34, *SD* = 6.08), and ordinary Internet users (class 4: 19.00%, *n* = 266, *M* = 69.83, *SD* = 9.55), respectively. Figure 1 displays the four classes scored in all the items. One-way ANOVA was performed for all four classes. There was a significant difference among the four classes. The *post-hoc* tests revealed that college students in one class significantly differed from those in another class in APIU scores (mean difference > 21.95, *p* < 0.001). In the *post-hoc* analyses, the class 1 subgroup scored significantly higher than classes 2, 3, and 4 subgroups on tolerance, mood alteration, social comfort, and negative outcomes (mean difference > 1.13, *p* < 0.001), respectively, while the class 2 subgroup scored significantly higher than classes 3 and 4 on tolerance, withdrawal symptoms, and social comfort (mean difference > 0.33, *p* < 0.05), respectively. There were no significant differences in the six dimensions of APIU between preferential and ordinary Internet users. However, preferential Internet users scored higher in terms of total APIU compared with ordinary Internet users (*p* < 0.001).

### Associations With Latent Classes

The statistical analyses of outcomes for the differences in class membership based on demographics, information gaining, Internet gaming, and communication preferences are shown in Table 3. Gender was not associated with the four classes of PIU. After accounting for gender, individuals with higher levels of Internet gaming and communication preferences were associated with increased odds of being in the pathological Internet users than that of the pathological-tendency Internet users, preferential Internet users, and ordinary Internet users. Individuals with higher levels of Internet gaming and communication preferences were associated with increased possibilities of being in the pathological-tendency Internet users than that of the preferential Internet users and ordinary Internet users. In addition, individuals with higher level of communication preferences were associated with increased possibilities of being in the preferential Internet users than that of ordinary Internet users.

**Table 3:** Multinomial logistic regressions predicting the Internet use classes from demographic characteristics, information gaining, Internet gaming, and communication variables with classes 1, 2, and 3 as reference groups.

	**Class 2 (vs. class 1)**		**Class 3 (vs. class 1)**		**Class 4 (vs. class 1)**		**Class 3 (vs. class 2)**		**Class 4 (vs. class 2)**		**Class 4 (vs. class 3)**	
	**B (SE B)**	**OR**	**B (SE B)**	**OR**	**B (SE B)**	**OR**	**B (SE B)**	**OR**	**B (SE B)**	**OR**	**B (SE B)**	**OR**
**Model 1**												
Gender	−0.07 (0.12)	0.93	0.02 (0.22)	1.03	0.23 (0.23)	1.26	0.09 (0.13)	1.10	0.30 (0.16)	1.35		
**Model 2**												
Gender	−0.10 (0.22)	0.91	−0.02 (0.22)	0.98	0.17 (0.24)	1.18	0.08 (0.14)	1.08	0.26 (0.16)	1.30	0.19 (0.16)	1.21
Information gaining	−0.01 (0.03)	0.95	0.014 (0.03)	1.01	0.017 (0.03)	1.02	0.02 (0.02)	1.02	0.03 (0.02)	1.03	0.002 (0.024)	1.002
Internet game	−0.05 (0.02)^**^	0.95	−0.10 (0.02)^**^	0.90	−0.10 (0.01)^**^	0.91	−0.05 (0.01)^**^	0.95	−0.05 (0.01)^**^	0.95	0.004 (0.013)	1.004
Communication	−0.13 (0.04)^**^	0.87	−0.20 (0.04)^**^	0.82	−0.27 (0.04)^**^	0.76	−0.07 (0.02)^**^	0.93	−0.14 (0.03)^**^	0.87	−0.07 (0.023)^**^	0.94

** *p < 0.01*.

## Discussion

The aim of this study was to comprehensively investigate whether IBPs (i.e., information gaining, Internet gaming, and communication preferences) are associated with the class membership which was obtained from the PIU sample of college students from a typological, person-centered perspective. These findings suggest that individuals can be classified into four Internet user profiles, namely, pathological Internet users (8.64%), pathological-tendency Internet users (36.43%), preferential Internet users (35.93%), and ordinary Internet users (19.00%), respectively. In comparison with pathological Internet users, lower Internet gaming and communication preference were associated with pathological-tendency users, preferential users, and ordinary Internet users.

In contrast with the earlier studies in which participants were categorized into Internet addictions and normal people (Morahan-Martin and Schumacher, 2000; Dong et al., 2014), this study further identified more specific classes according to the different tendencies of the PIU. Although the class of Internet users has become a debate topic in recent studies, a consistent conclusion has yet to be reached. For instance, Mok et al. (2014) reported that the three-class model is the best model, whereas Rumpf et al. (2014) opted for the six-class model. In this study, we considered the four-class model owing to the following reasons: first, individuals at a different range of development stages may fit different class models. Specifically, this study focused on the individuals aged 17–25 years old; however, earlier studies chose the individuals aged 13–16 years (Van Rooij et al., 2011) or 14–46 years (Rumpf et al., 2014) as the targeted populations. Second, different measurement tools were used in different studies. Earlier studies measured Internet addiction by using the compulsive Internet use scale or IAT (Mok et al., 2014; Rumpf et al., 2014; Hussain et al., 2020) while the APIU was utilized in this study. Finally, different cultures may have a different impact on the model classification. A meta-analysis study revealed that cultural characteristics play a different role in the mechanisms of Internet addiction (Cheng et al., 2018). Earlier studies had been demonstrated the results obtained from various studies conducted in Germany (Rumpf et al., 2014), Korea (Mok et al., 2014), and Pakistan (Hussain et al., 2020), whereas this study was conducted in China. Similarly, concerning the uniqueness of the Chinese culture, the classes of Internet use may differ from those mentioned in earlier studies. Therefore, future studies should adopt the cross-cultural paradigm design to get more generalized and specific results of PIU.

The proportion of PIU was the smallest among all the classes, which conformed to earlier studies (Mok et al., 2014; Rumpf et al., 2014). Moreover, 8.64% of the pathological Internet users who participated in this study are similar to those who participated in a meta-analysis study, which involved 31 nations across seven world regions and find that there was 6% of Internet addiction in all the participants (Cheng and Li, 2014). Based on the users and gratification theory (Palmgreen et al., 1985), pathological-tendency Internet users may employ the Internet to avoid reality, obtain information, or entertain themselves (Raacke and Bonds-Raacke, 2008; Smock et al., 2011). Moreover, people are susceptible to the availability of the Internet. Earlier studies revealed that individuals who had a high frequency of using smartphones were more addicted to these devices (Salehan and Negahban, 2013; Van Deursen et al., 2015). For the PIU, there is a highly similar developmental pattern between Internet addiction and smartphone addiction. Therefore, it is crucial to develop a series of initiatives to curb and alleviate the problem in pathological-tendency Internet users, such as reducing the time spent on the Internet and the intensity of Internet use. In addition, there existed a higher score in terms of total APIU in the preferential Internet users compared with ordinary Internet users, suggesting a quantitative difference in processing time and intensity of Internet use between them. The relationship between the two types of Internet users needs further clarification in similarities and differences.

The four classes identified by LPA were not diverse with regard to gender, suggesting that males were not at increased possibilities of being pathological Internet users as compared with female users. After controlling for gender, different IBP dimensions were associated significantly with class membership. Information gaining, Internet gaming, and communication preferences are different across the classes, indicating that these individual difference factors may play a role in initiation and continuation of pathological Internet users. Information gaining preference was not associated with all the four classes. Internet gaming and communication preferences in this study could predict PIU, which was consistent with the earlier studies. PIU can be positively predicted cross-sectionally by gaming preference (Meerkerk et al., 2006; Van Rooij et al., 2010; Rehbein and Mößle, 2013) and Internet social contacts (Luo et al., 2010). On the one hand, the Internet has a characteristic of anonymity, convenience, and evasion, which allows college students to escape from real-life stress and gain a sense of accomplishment, belongingness, or gratification (Luo et al., 2010). On the other hand, the more time college students spend on the Internet, the more self-efficacy they derive from it, which eventually leads to PIU (Tella, 2011). Therefore, surfing online for a long time may account for the high levels of addiction in college students.

However, this study showed that information gaining preference was not associated with PIU, which is consistent with the earlier studies (Leung, 2004; Lei et al., 2006). The reason could be that gaining information, including browsing the web and downloading information, has become a normal Internet use behavior [China Internet Network Information Center (CNNIC), 2020]. These online activities may provide only a limited feeling of pleasure and hence hardly drive college students to invest considerable time in Internet usage. Besides, accessing to information mainly aims to attain a realistic purpose, which may explain why information gaining preference alone unlikely leads to Internet addiction among college students (Lei et al., 2006). Assuming that changes in IBP translate to a decrease in Internet addictions, grouping individuals together into classes of PIU patterns may hold promise for tailoring interventions to PIU prevention. Thus, it may be important for colleges to take different measures of prevention and intervention programs in place to tailor treatment to specific types of emerging adults. There may also be utilized in pretesting college students and using this information to appropriately target Internet use prevention and intervention programs.

Although this study provides valuable insight into the complexity of Internet use behaviors among a sample of emerging college students, the results must be interpreted in light of some important considerations. First, the small pathological Internet user group (8.64%) is compatible with the psychological and criminological research that recurrently finds evidence of a small pathological Internet user group that engages in many problem behaviors (Vaughn et al., 2011, 2014). However, there are no additional data to examine the association between PIU and problem behaviors in this study. It would be of added value if future studies observe the association between PIU and problem behaviors. Second, as this study focused on college students, future research would examine predictors of PIU in different samples. For example, given that the PIU of those who attend college may differ from those who do not, a broad range of ages in university-attending and non-university-attending emerging adults should be included. In addition, considering the different severity of PIU, clinical samples with Internet addiction should also be explored. Third, in addition to the method of self-report, our findings should be corroborated by other methods, such as event-related potential to extend our understanding of the mechanism of PIU. Finally, although our research was performed by using the one-way ANOVA to distinguish the four groups by the total score of APIU, additional covariates are also needed to collect in order to further verify the profile we classified.

The present results highlight an important role of IBP in PIU among college students. The focus has recently been toward preventing the uptake of PIU among adolescents; however, given the high prevalence of Internet initiation among college students, more potential prevention and intervention techniques are needed to be developed specifically for college students. Since IBP are amenable to change, it is possible to tailor prevention to those who are most at risk for PIU.

## Data Availability Statement

The original contributions presented in the study are included in the article/supplementary material, further inquiries can be directed to the corresponding author.

## Ethics Statement

The studies involving human participants were reviewed and approved by Beijing Normal University. The patients/participants provided their written informed consent to participate in this study.

## Author Contributions

GZ contributed ideas, collected and analyzed the data, and wrote the article. JC and JL collected and analyzed the data and wrote the article. FK revised the article. All the authors contributed to the article and approved the submitted version.

## Conflict of Interest

The authors declare that the research was conducted in the absence of any commercial or financial relationships that could be construed as a potential conflict of interest.

## References

[B1] Akaike H. (1974). A new look at the statistical model identification. IEEE Trans. Automat. Contr. 19, 716–723. 10.1007/978-1-4612-1694-0_16

[B2] Arafa A. Mahmoud O. Salem E. A. (2019). Excessive internet use and self-esteem among internet users in Egypt. Int. J. Ment. Health. 48, 95–105. 10.1080/00207411.2019.1611167

[B3] Burnham K. P. Anderson D. R. (2004). Multimodel inference understanding AIC and BIC in model selection. Sociol. Method Res. 33, 261–304. 10.1177/0049124104268644

[B4] Caplan S. E. (2002). Problematic Internet use and psychosocial well-being: development of a theory-based cognitive–behavioral measurement instrument. Comput. Hum. Behav. 18, 553–575. 10.1016/s0747-5632(02)00004-3

[B5] Chen S. H. Weng L. J. Su Y. J. Wu H. M. Yang P. F. (2003). Development of a Chinese internet addiction scale and its psychometric study. Chin. J. Psychol. 45, 279–294. 10.1037/t44491-000

[B6] Cheng C. Cheung M. W. Wang H. (2018). Multinational comparison of internet gaming disorder and psychosocial problems versus well-being: meta-analysis of 20 countries. Comput. Hum. Behav. 88, 153–167. 10.1016/j.chb.2018.06.033

[B7] Cheng C. Li A. Y. L. (2014). Internet addiction prevalence and quality of (real) life: a meta-analysis of 31 nations across seven world regions. Cyberpsychol. Behav. Soc. Netw. 17, 755–760. 10.1089/cyber.2014.0317 25489876 PMC4267764

[B8] China Internet Network Information Center (CNNIC) (2020). Available online at: http://www.cnnic.cn/gywm/xwzx/rdxw/202009/t20200929_71255.htm (accessed September 29, 2020).

[B9] Davis R. A. (2001). A cognitive-behavioral model of pathological internet use. Comput. Hum. Behav. 17, 187–195. 10.1016/S0747-5632(00)00041-8

[B10] De Leo J. A. Wulfert E. (2013). Problematic Internet use and other risky behaviors in college students: an application of problem-behavior theory. Psychol. Addict. Behav. 27, 133–141. 10.1037/a0030823 23276311

[B11] Dieter J. Hoffmann S. Mier D. Reinhard I. Beutel M. Vollstädt-Klein S.. (2017). The role of emotional inhibitory control in specific Internet addiction–an fMRI study. Brain Behav. Res. 324, 1–14. 10.1016/j.bbr.2017.01.046 28174031

[B12] Dong G. Lin X. Zhou H. Lu Q. (2014). Cognitive flexibility in internet addicts: fMRI evidence from difficult-to-easy and easy-to-difficult switching situations. Addict. Behav. 39, 677–683. 10.1016/j.addbeh.2013.11.028 24368005

[B13] Forrest C. J. King D. L. Delfabbro P. H. (2016). The measurement of maladaptive cognitions underlying problematic video-game playing among adults. Comput. Hum. Behav. 55, 399–405. 10.1016/j.chb.2015.09.017

[B14] Hussain I. Cakir O. Ozdemir B. (2020). Studying internet addiction profile of university students with latent class analysis. Educ. Inf. Technol. 25, 4937–4959. 10.1007/s10639-020-10203-6

[B15] International Telecommunication Union (ITU) (2018). Measuring the Information Society Report 2018. Available online at: https://www.itu.int/en/ITU-D/Statistics/Documents/publications/misr2018/MISR-2018-Vol-1-E.pdf (accessed December 10, 2018).

[B16] Kemp S. (2019). Digital 2019: Global Internet Use Accelerates. Available online at: https://wearesocial.com/blog/2019/01/digital-2019-global-internet-use-accelerates (accessed January 30, 2020).

[B17] Koronczai B. Kökönyei G. Griffiths M. D. Demetrovics Z. (2019). The relationship between personality traits, psychopathological symptoms, and problematic internet use: a complex mediation model. J. Med. Internet Res. 21:e11837. 10.2196/11837 31025955 PMC6658222

[B18] Kuss D. J. Griffiths M. D. (2011). Online social networking and addiction—a review of the psychological literature. Int. J. Environ. Res. Public Health 8, 3528–3552. 10.3390/ijerph8093528 22016701 PMC3194102

[B19] Lee S. Y. Lee D. Nam C. R. Kim D. Y. Park S. Kwon J. G.. (2018). Distinct patterns of internet and smartphone-related problems among adolescents by gender: Latent class analysis. J. Behav. Addict. 7, 454–465. 10.1556/2006.7.2018.28 29788762 PMC6174601

[B20] Lei L. Yang Y. (2007). The development and validation of adolescent pathological Internet use scale. Acta Psychol. Sin. 39, 688–696. 10.1037/t53624-000

[B21] Lei L. Yang Y. Liu M. (2006). The relationship between adolescents' neuroticism, internet service preference, and internet addiction. Acta Psychol. Sin. 38, 375–381.

[B22] Leung L. (2004). Net-generation attributes and seductive properties of the Internet as predictors of online activities and Internet addiction. Cyberpsychol. Behav. 7, 333–348. 10.1089/1094931041291303 15257834

[B23] Li J. B. Wu A. M. S. Feng L. F. Deng Y. Lau J. T. F. (2020). Classification of probable online social networking addiction: a latent profile analysis from a large-scale survey among Chinese adolescents. J. Addict. Behav. 9, 698–708. 10.1556/2006.2020.00047 32829311 PMC8943659

[B24] Li S. M. Chung T. M. (2006). Internet function and Internet addictive behavior. Comput. Hum. Behav. 22, 1067–1071. 10.1016/j.chb.2004.03.03

[B25] Li X. Y. Zheng X. F. (2010). A study on the attentional bias to emotional information in pathological Internet user. Psycho. Dev. Educ. 26, 357–363.

[B26] Lo Y. Mendell N. R. Rubin D. B. (2001). Testing the number of components in a normal mixture. BIOMETRIKA 88, 767–778. 10.1093/biomet/88.3.767

[B27] Luo J. H. Wan J. J. Liu X. Q. Fang X. Y. (2010). The relationship of internet use, internet special self-efficacy, and internet addiction in university students. Psychol. Dev. Educ. 26, 618–626.

[B28] Meerkerk G. J. Eijnden R. J. V. D. Garretsen H. F. (2006). Predicting compulsive Internet use: it's all about sex! Cyberpsychol. Behav. 9, 95–103. 10.1089/cpb.2006.9.95 16497122

[B29] Meerkerk G. J. Van Den Eijnden R. J. Vermulst A. A. Garretsen H. F. (2009). The compulsive Internet use scale (CIUS): some psychometric properties. Cyberpsychol. Behav. 12, 1–6. 10.1089/cpb.2008.0181 19072079

[B30] Mok J. Y. Choi S. W. Kim D. J. Choi J. S. Lee J. Ahn H.. (2014). Latent class analysis on Internet and smartphone addiction in college students. Neuropsychiatr. Dis. Treat. 10, 817–827. 10.2147/NDT.S59293 24899806 PMC4038421

[B31] Morahan-Martin J. Schumacher P. (2000). Incidence and correlates of pathological internet use among college students. Comput. Hum. Behav. 16, 13–29. 10.1016/S0747-5632(99)00049-7

[B32] Morin A. J. Morizot J. Boudrias J. S. Madore I. (2011). A multifoci person-centered perspective on workplace affective commitment: a latent profile/factor mixture analysis. Organ. Res. Methods 14, 58–90. 10.1177/1094428109356476

[B33] Muthén L. K. Muthén B. O. (1998–2015). Mplus User's Guide. 7th Edn. Los Angeles, CA: Muthén and Muthén.

[B34] Nylund K. L. Asparouhov T. Muthén B. O. (2007). Deciding on the number of classes in latent class analysis and growth mixture modeling: a Monte Carlo simulation study. Struct. Equ. Modeling 14, 535–569. 10.1080/10705510701575396

[B35] Palmgreen P. Wenner L. Rosengren K. (1985). Media gratifications research, in The Uses and Gratifications Research: The Past Ten Years, eds Wenner Palmgreen P. (Beverly Hills, CA: Sage), 11–37.

[B36] Raacke J. Bonds-Raacke J. (2008). MySpace and facebook: applying the uses and gratifications theory to exploring friend-networking sites. Cyberpsychol. Behav. 11, 169–174. 10.1089/cpb.2007.0056 18422409

[B37] Rehbein F. Mößle T. (2013). Video game and Internet addiction: is there a need for differentiation? Sucht 59, 129–142. 10.1024/0939-5911.a000245

[B38] Rumpf H. J. Vermulst A. A. Bischof A. Kastirke N. Gürtler D. Bischof G.. (2014). Occurence of Internet addiction in a general population sample: a latent class analysis. Eur. Addict. Res. 20, 159–166. 10.1159/000354321 24401314

[B39] Salehan M. Negahban A. (2013). Social networking on smartphones: when mobile phones become addictive. Comput. Hum. Behav. 29, 2632–2639. 10.1016/j.chb.2013.07.003

[B40] Schehl B. Leukel J. Sugumaran V. (2019). Understanding differentiated internet use in older adults: a study of informational, social, and instrumental online activities. Comput. Hum. Behav. 97, 222–230. 10.1016/j.chb.2019.03.031

[B41] Schwarz G. (1978). Estimating the dimension of a model. Ann. Stat. 6, 461–464. 10.1214/aos/1176344136

[B42] Shao Y. J. Zheng T. Wang Y. Q. Liu L. Chen Y. Yao Y. S. (2018). Internet addiction detection rate among college students in the people's republic of china: a meta-analysis. Child. Adolesc. Psychiatry Ment. Health 12:25. 10.1186/s13034-018-0231-6 29849754 PMC5970523

[B43] Shin S. H. McDonald S. E. Conley D. (2018). Patterns of adverse childhood experiences and substance use among young adults: a latent class analysis. Addict. Behav. 78, 187–192. 10.1016/j.addbeh.2017.11.020 29179155 PMC5783745

[B44] Siomos K. E. Dafouli E. D. Braimiotis D. A. Mouzas O. D. Angelopoulos N. V. (2008). Internet addiction among Greek adolescent students. CyberPsychol. Behav. 11, 653–657. 10.1089/cpb.2008.0088 18991535

[B45] Smock A. D. Ellison N. B. Lampe C. Wohn D. Y. (2011). Facebook as a toolkit: a uses and gratification approach to unbundling feature use. Comput. Hum. Behav. 27, 2322–2329. 10.1016/j.chb.2011.07.011

[B46] Sun X. J. Zhao J. Zhou Z. K. Chen W. Bao N. (2015). Mediation role of self-control in Internet use between time management disposition and pathological Internet use. Stud. Psychol. Behav. 13, 410–413.

[B47] Tella A. (2011). An assessment of mathematics teachers' internet self-efficacy: implications on teachers' delivery of mathematics instruction. Int. J. Math. Educ. Sci. Technol. 42, 155–174. 10.1080/0020739X.2010.519798

[B48] Tian Y. Guo Z. X. Shi J. R. Bian Y. L. Han P. G. Wang P.. (2018). Bidirectional mediating role of loneliness in the association between shyness and generalized pathological internet use in Chinese university students: a longitudinal cross-lagged analysis. J. Psychol. 152, 529–547. 10.1080/00223980.2018.1468309 30376647

[B49] Van Deursen A. J. Bolle C. L. Hegner S. M. Kommers P. A. (2015). Modeling habitual and addictive smartphone behavior: the role of smartphone usage types, emotional intelligence, social stress, self-regulation, age, and gender. Comput. Hum. Behav. 45, 411–420. 10.1016/j.chb.2014.12.039

[B50] Van Rooij A. J. Schoenmakers T. M. Van de Eijnden R. J. Van de Mheen D. (2010). Compulsive Internet use: the role of online gaming and other Internet applications. J. Adolesc. Health 47, 51–57. 10.1016/j.jadohealth.2009.12.021 20547292

[B51] Van Rooij A. J. Schoenmakers T. M. Vermulst A. A. Van Den Eijnden R. J. Van De Mheen D. (2011). Online video game addiction: identification of addicted adolescent gamers. Addiction 106, 205–212. 10.1111/j.1360-0443.2010.03104.x 20840209

[B52] Vaughn M. G. DeLisi M. Gunter T. Fu Q. Beaver K. M. Perron B. E.. (2011). The severe 5%: a latent class analysis of the externalizing behavior spectrum in the United States. J. Crim. Justice 39, 75–80. 10.1016/j.jcrimjus.2010.12.001 22942480 PMC3431912

[B53] Vaughn M. G. Salas-Wright C. P. DeLisi M. Maynard B. R. (2014). Violence and externalizing behavior among youth in the United States: is there a severe 5%? Youth Violence Juv. Justice 12, 3–21. 10.1177/1541204013478973

[B54] Wang L. Shen H. Lei Y. Zeng L. L. Cao F. Su L.. (2017). Altered default mode, fronto-parietal, and salience networks in adolescents with Internet addiction. Addict. Behav. 70, 1–6. 10.1016/j.addbeh.2017.01.021 28160660

[B55] Young K. S. (1998). Internet addiction: the emergence of a new clinical disorder. Cyberpsychol. Behav. 1, 237–244. 10.1089/cpb.1998.1.237

[B56] Young K. S. Abreu C. (2011). Internet Addiction. A Handbook and Guide to Evaluation and Treatment. Hoboken, NJ: John Wiley and Sons.

[B57] Zheng X. Gu H. (2013). Relationship between Internet altruistic behavior of undergraduates and internet-behavior preference: effects of class environments. Stud. Psychol. Behav. 11, 690–696.

[B58] Zhou L. Gu H. (2008). A research on the Internet-behaviors preferences of undergraduates in shanghai. Psychol. Sci. 31, 1353–1356. 10.16719/j.cnki.1671-6981.2008.06.024

